# Ancient DNA studies performed in the forensic genetic laboratory in Slovenia: a narrative review

**DOI:** 10.3325/cmj.2026.67.30

**Published:** 2026-02

**Authors:** Irena Zupanič Pajnič

**Affiliations:** Institute of Forensic Medicine, Faculty of Medicine, University of Ljubljana, Ljubljana, Slovenia

## Abstract

Ancient DNA research is typically conducted in dedicated clean-room facilities; however, recent work in Slovenia has demonstrated that standard forensic technologies can also be successfully applied to archaeological remains. This article reviews studies conducted by our group on skeletal samples from different Slovenian sites. Comparative analyses of petrous bones from two archaeological contexts revealed how environmental conditions affect DNA preservation. Long-term storage effects were assessed by comparing freshly excavated remains with samples stored for 12 years under unregulated museum conditions. Intra-skeletal variability was examined through petrous bones, femurs, tali, calcanei, patellae, and teeth, and the potential of tooth cementum for minimally destructive extraction was evaluated. Differences in DNA quality between adult and non-adult skeletons were investigated. Genetic sexing was performed on 83 subadult skeletons to test the reliability of morphological methods, which often fail at this age. For optimized analyses, a DNA extraction protocol was developed that requires minimal bone powder. The PowerQuant real-time polymerase chain reaction (PCR) system proved a cost-effective predictor of successful short tandem repeat (STR) typing in degraded remains. Kinship studies, including that of four individuals from the 5th-6th century, demonstrated the utility of combining STR, single nucleotide polymorphism (SNP), and PCR-based massive parallel sequencing approaches, which improved statistical power and confirmed relatedness. Additionally, eye and hair color were predicted for skeletons dated to the 3rd-18th centuries. Overall, these results highlight that combining conventional forensic approaches with strict contamination control can generate reliable genetic data from archaeological samples and offer valuable insights into past populations.

Ancient DNA (aDNA) refers to genetic material recovered from poorly preserved remains that are often centuries or thousands of years old. The threshold separating archaeological from forensic human remains varies internationally, with many countries designating material older than 50-100 years as archaeological (1). Other factors, such as the feasibility of identifying the individual, also influence classification. If a suspect is still alive and legal action is possible, the remains are treated as forensic, which defines aDNA as DNA older than 70 years post-mortem (2).

Both forensic DNA investigations and aDNA research face similar obstacles, including the scarcity and poor preservation of genetic material, the need for rigorous measures to prevent contamination, and the application of verification criteria (3). While aDNA studies once relied on polymerase chain reaction (PCR)-based methods, recent advances have introduced high-throughput approaches. Despite their power, these modern techniques are considerably more expensive than conventional forensic methods and require specialized facilities and expertise. Considering this, our research group performed several aDNA analyses using established forensic tools. This article reviews studies conducted by our group on skeletal samples from different Slovenian sites.

Ancient skeletal remains typically contain only trace amounts of endogenous DNA, which is often severely degraded and highly susceptible to contamination with modern human DNA (4). The reliability of genetic investigations depends critically on appropriate procedures during excavation, anthropological processing, and long-term storage. Improper handling can accelerate the degradation of endogenous DNA and introduce exogenous contamination (5). To ensure data authenticity, three principal considerations must be observed. First, the use of protective equipment is mandatory. Second, cleaning procedures must consider that washing bones before storage adversely affects DNA preservation (6). Third, skeletal material should be stored under conditions that prevent further molecular damage. Fulton (7) emphasized that optimal long-term storage depends on the environmental context of recovery. In general, avoiding conditions known to accelerate DNA damage is essential, with stable, cold, and dry environments being most suitable (7,8).

Surface contamination is a frequent obstacle. Depending on porosity, contaminating DNA may infiltrate beyond the surface layers of bone and teeth (9,10). Various decontamination procedures are therefore employed, including mechanical removal of the bone surface and targeting DNA extraction from the inner bone or tooth material (11).

Contamination may also arise during laboratory procedures from handling without protective gear or from exogenous DNA carried by reagents, laboratory materials, or airborne aerosols (12). Given the fragmented nature of aDNA, the risk remains exceptionally high (5,12). Consequently, laboratory practice must emphasize prevention, detection, and rigorous authentication (5,11). Key indicators of authenticity include the inverse relationship between amplification efficiency and fragment length, damage patterns, and characteristic nucleotide misincorporations (5).

Best practices require that samples be processed in dedicated pre-PCR facilities (11). Such facilities should be equipped with HEPA-filtered clean benches. Laboratory surfaces must be routinely decontaminated, and personnel should wear full protective clothing (5). Moreover, an elimination database containing genetic profiles of all individuals who have come into contact with the samples is essential. Negative controls should accompany every batch of samples (5). Finally, independent DNA extractions from the same specimen must be performed to confirm reproducibility (5).

In all our research, precautions described above were followed, and authenticity was checked through quantitative PCR degradation rate determination and short tandem repeat (STR) typing. Matched profiles across different skeletal elements, contamination controls, and elimination databases also served for confirmation.

In most of our research, petrous bones were sampled. They are among the best sources for aDNA (13-15) due to their resistance to degradation, high DNA preservation capacity (13,16), high density, low remodeling rate, and high osteocyte concentration (17-19).

In all our research, DNA preservation was assessed through quantitative real-time PCR using the PowerQuant System (Promega, Madison, WI, USA) to assess the concentration of nuclear DNA and DNA degradation. For STR typing, the PowerPlex ESI 17 Fast System (Promega) was used. DNA was extracted using a high-efficiency protocol (20).

## Effect of different environmental factors and storage conditions on DNA preservation

Kravanja et al investigated how environmental conditions influence DNA preservation in petrous bones by comparing two Slovenian archaeological sites: Ljubljana – Njegoševa and Črnomelj (21). Njegoševa has an alkaline soil pH (7.4-7.5), while Črnomelj has acidic soil (pH 5.2-6). The climatic conditions also differ slightly, with Črnomelj being warmer and wetter (21). Higher temperatures in Črnomelj likely accelerated DNA degradation by enhancing hydrolytic damage and microbial activity (5). Acidic soil may have contributed to the dissolution of hydroxyapatite, destabilizing the DNA-mineral complex (22). In contrast, the alkaline soil in Njegoševa likely contributed to better DNA preservation (23). Although Njegoševa had higher organic matter, no significant inhibition was detected, which suggests successful purification (24). The study demonstrated that environmental variables, particularly soil pH, temperature, and water permeability, played critical roles in aDNA preservation.

Jeromelj et al evaluated the impact of long-term storage on nuclear DNA preservation, comparing freshly excavated samples to those stored for 12 years in unregulated museum conditions (25). The study emphasized that although DNA can survive for thousands of years in favorable burial environments, post-excavation changes can significantly accelerate degradation (26). DNA yield was significantly higher in freshly excavated bones, while the degradation index was higher in stored samples. These findings suggest that unregulated temperature and humidity in museum depots may contribute to DNA decay, even in dense bone structures like the petrous portion (6). After excavation, fluctuations in temperature and humidity disrupt the equilibrium established underground, accelerating biochemical decay (27). While cold storage is often employed, repeated freeze-thaw cycles may negatively affect DNA quality (28). We recommend maintaining stable room temperatures (16-20°C) and relative humidity (45-65%) as a feasible compromise (25). Institutions should also avoid daily or seasonal fluctuations and protect samples from UV exposure, as this can induce DNA mutations and reduce bone density (29). Our results support previous research (30) indicating that skeletal remains maintain better DNA quality when buried.

## Intra-skeletal variability in DNA preservation

Golob et al examined DNA preservation by comparing petrous bones to femurs, tali, and calcanei from 66 adult skeletons from two Slovenian sites: Črnomelj (13th to 18th century) and Polje (16th to 19th century) (31). The motivation was the need for alternative skeletal elements suitable for DNA analysis when petrous bones are unavailable (13,14,32). DNA quantification and STR typing success were assessed. Results showed significant differences across bones and between sites. In Črnomelj, the petrous bone outperformed other elements, with the calcaneus being the only bone matching its performance. However, at the Polje site, no significant difference was observed, which suggests that tali, calcanei, and femurs are viable alternatives in more recent contexts. The higher STR typing success in Polje is attributed to shorter post-mortem intervals and more favorable preservation conditions (33). Trabecular bones are more prone to decay due to higher porosity (34), but their ability to retain soft tissue remnants may support higher DNA yields under favorable conditions (35-38). DNA degradation was consistently higher in petrous bones. Despite this, they still produced high STR typing success due to their dense structure, low remodeling rate, and reduced vascularization (39). The high osteocyte density in petrous bones further supports superior DNA retention (13).

## Tooth cementum and patellae as a source of aDNA

Zupanič Pajnič at al explored the suitability of tooth cementum as a reliable DNA source compared with petrous bones (40). DNA quantity, degradation rate, and STR typing success in 60 adult archaeological skeletons were assessed. Petrous bones yielded significantly more DNA but exhibited higher degradation indices. However, all petrous bone samples and 45 of 60 teeth produced highly informative STR profiles. When poorly preserved teeth were excluded, no significant difference was found in STR typing success. This demonstrates that when well-preserved, teeth perform as well as petrous bones. The higher degradation in petrous bones was possibly caused by mechanical stress during grinding (20). In contrast, the tooth cementum extraction used a nondestructive method that reduced the risk of DNA damage, aligning with ethical conservation (41,42). Tooth cementum has practical advantages. Cementocytes contain abundant DNA (43). Cementum thickness increases with age, improving DNA recovery (44). Teeth are less porous, more resistant to environmental degradation, and often better preserved than bones (45).

Geršak et al explored the viability of patellae as a DNA source by comparing samples from a post-Second World War mass grave and a 13th-19th century cemetery (46). Patellae are sesamoid bones with a high proportion of cancellous tissue (38). DNA yields were significantly higher in Second World War patellae. Similarly, the degradation index was notably lower, reflecting better preservation. STR typing produced full profiles in 90% of Second World War patellae and informative partial profiles in 52% of archaeological samples. The cancellous nature of patellae makes them vulnerable to decay over longer periods (37). Their distinct anatomy aids identification. The results confirm that patellae are a promising alternative to traditional bones, particularly when standard bones are fragmented or absent (46,47).

## Bone and tooth DNA preservation in adult and non-adult skeletal remains

Šuligoj et al compared DNA preservation between adult and non-adult remains, focusing on the petrous bone, femur, calcaneus, and talus (48). Fifty-two skeletons (29 adults and 23 non-adults) were analyzed. The petrous bone was confirmed as the most reliable element for non-adults, providing higher yields and better STR typing success than other bones, possibly due to lower degradation (15,49). In adults, the talus, alongside the petrous bone, yielded high-quality STR profiles, while the femur showed poor preservation. DNA yield was higher in adult bones overall, but STR typing success was better in non-adult than in adult petrous bones. No significant differences were found between sexes or between age subgroups in non-adults. The poorer preservation in femurs and foot bones of non-adults is attributed to their porosity and smaller size (50,51). Adult tali and calcanei performed better than non-adult, possibly due to increased bone remodeling (52).

Leskovar et al examined DNA preservation in different tooth types from adults and non-adults (53). Adults' teeth yielded significantly more DNA than non-adults’. Permanent teeth produced higher yields than deciduous ones. Fully developed permanent teeth outperformed their not fully developed counterparts. STR typing reflected this disparity. Pathology did not significantly impact DNA preservation. Cementum, which thickens with age, likely contributes to higher DNA yields in adult teeth (49,54).

## Genetic and morphological methods for sex determination of subadult skeletons

Zupanič Pajnič et al performed genetic sexing on petrous bones from 83 subadult skeletons using three molecular techniques: the qPCR PowerQuant Y-target test, the amelogenin test, and the PowerPlex Y-23 kit (55). These methods successfully identified the sex of 78/83 skeletons. The otic capsule of the petrous bone was the preferred sample, and even newborns and stillborn infants provided DNA enabling sex identification.

In the study by Leskovar et al comparing morphological and genetic sex assessment on 116 skeletons, accuracy differed greatly (56). Morphological methods correctly assessed the sex of 97% of adults, but only of 72% of non-adults. This reflects the unreliability of morphological sex assessment in subadult remains. Genetic sex determination was highly successful, identifying sex in 109 of 116 individuals. The pelvis was the most reliable element for morphological assessment (57). All three molecular tests were critical for confirming male sex.

## DNA extraction methods from skeletal remains

The study by Zupanič Pajnič et al on extraction methods presented an optimized protocol for small quantities of bone powder (58). A modified protocol using 75 mg of bone powder and a shortened decalcification time was tested against a standard method. The new protocol was effective for relatively well preserved ancient samples, yielding comparable results. However, the standard protocol was more effective in more degraded samples. The standard protocol's use of EDTA is crucial for complete demineralization, especially in older samples (10,11,59). However, the optimized protocol offers several advantages: it reduces the required sample, minimizes reagent preparation, and allows high-throughput processing, thus reducing contamination risk.

The study by our team on a nondestructive DNA extraction method presented a novel extraction from the cementum layer of tooth roots (60). The method proved efficient, with 74% of samples yielding informative STR profiles. Its strength lies in minimal invasiveness and reduced contamination risk. About one-quarter of canines failed, largely due to low DNA yield influenced by environmental conditions (61,62). The method shows strong potential for applications where preserving the specimen is paramount.

## Prediction of STR typing success using quantitative PCR

In 2017, our team evaluated the PowerQuant qPCR system (Promega) to predict STR typing success in degraded skeletal remains (63). The key predictive factor was a simultaneous detection of both short (Auto, 85 bp) and long (Deg, 294 bp) autosomal targets (64). Samples where only the Auto target or no targets were detected failed. A simplified model based on the detection of both targets correctly predicted the outcome in 85% of cases. STR typing can succeed even when DNA concentrations are below the detection threshold, as long as the Deg target is amplified (63). Using PowerQuant for pre-screening helps avoid unnecessary STR attempts on samples unlikely to succeed, conserving resources.

## Eye and hair color prediction

In 2022, we evaluated a forensic PCR-massively parallel sequencing (MPS) approach for eye and hair color prediction in aged remains (65). A customized HIrisPlex panel (Erasmus University Medical Center, Rotterdam, the Netherlands) was used on 24 SNPs. Consensus genotypes enabled predictions for 10 out of 11 ancient skeletons. The method is viable for ancient samples, provided that multi-sample consensus genotyping is employed and contamination safeguards are implemented (5,65).

A 2023 study by our research group on an early medieval adult and a subadult successfully demonstrated phenotyping from a subadult individual (66). Both individuals yielded high quantities of DNA. Sequencing metrics were even higher in the subadult sample. Predicted phenotypes indicated brown eyes/dark hair for the adult and blue eyes/brown hair for the subadult. Excellent DNA preservation in the subadult sample emphasizes the utility of the petrous bone (13,15).

## Kinship analysis

Another study by our team investigated genetic relationships among four individuals from a 5th-6th-century shared grave (67). Autosomal STR typing revealed nearly complete profiles. Statistical analysis confirmed the adult man as the father of the other three individuals. Y-haplogroup prediction assigned both men to the E1b1b haplogroup. The study highlights the importance of petrous bone sampling over teeth in aDNA recovery, particularly for subadults (50).

We also presented a case where STR analysis did not provide sufficient statistical support. Identity SNP analysis using a PCR-MPS approach was integrated to improve kinship probability (68). The SNP data produced a high likelihood ratio. Combining STR and SNP results confirmed full-sibling kinship. This shows that identity SNPs offer higher resolution and are less affected by aDNA degradation (19,69).

## Discussion

This review shows that the forensic approach can be effectively applied to some analyses of ancient skeletal remains. Low quantity and high degradation of aDNA presents major challenges – especially due to the risk of contamination with modern human DNA. To address these issues, studies included in this review followed strict protocols designed to prevent contamination and ensure the authenticity of aDNA results.

Environmental factors profoundly affect DNA preservation. Key determinants include soil composition, pH, moisture, microbial activity, and temperature (50,70,71). Optimal preservation occurs under conditions of minimal UV exposure, rapid desiccation, low humidity, and low temperature (6). Our studies on different sites (21) and storage conditions (25) confirmed this significant influence. Storage conditions are crucial for museums, archaeological institutions, and forensic laboratories that handle ancient or historical bone material. Sampling should ideally occur shortly after excavation, and when not possible, storage environments must adhere to current best-practice guidelines to minimize further degradation of valuable genetic material.

Genetic research involving archaeological skeletal remains is often limited by the incomplete preservation of bones, which are also considered valuable cultural heritage. As genetic analysis is destructive – requiring bone or tooth material to be ground into powder – it must be well justified, ensuring value both to genetics and heritage preservation. Thus, it is crucial to identify the most suitable elements for aDNA extraction and explore viable alternatives. Because skeletal elements differ in DNA preservation (72), peatellae (46), tooth cementum (60), and petrous bones (40) were studied. In addition, petrous bones were compared with femurs, tali, and calcanei (31). Patellae offer a reliable source, calcanei and tali can serve as valuable alternatives, and tooth cementum is a viable and often preferable sample type, particularly with a nondestructive method (31,40,46,60). Comparison of different skeletal elements between adult and non-adult skeletons showed that only petrous bones should be considered for DNA sampling in non-adults, whereas in adults, petrous bones and tali are both reliable (48). For teeth, fully developed permanent teeth from adults should be prioritized (53).

Accurate sexing of subadult skeletons is vital for understanding demographic structures of past populations and for identification in forensic scenarios. Genetic sexing of 83 subadults demonstrated that, with appropriate DNA methods, subadult sexing can be highly successful, regardless of age. It also established petrous bone sampling as essential for future studies of degraded subadult remains (55). A combination of morphological and genetic methods yielded the most reliable sex determinations (56).

Highly efficient extraction methods are crucial for successful DNA typing of skeletal remains. Our optimized protocol for small quantities is suitable for relatively well-preserved ancient remains, while for highly degraded remains, full demineralization remains necessary (58). The nondestructive method from tooth cementum represents a significant advancement (60).

The PowerQuant system (Promega) serves as a reliable pre-screening tool for predicting STR typing success, helping to reduce costs and conserve DNA extract (63). The PCR-MPS HIrisPlex method is a viable option for predicting eye and hair color in ancient remains, and consensus genotyping remains essential (65,66). Kinship analysis using modern forensic techniques provides strong evidence of familial burial practices (67). When STR-based analysis fails, identity SNPs provide a powerful complementary tool (68).

### Conclusions

In this review, we concentrated on molecular genetic analyses of aDNA performed in a forensic genetic laboratory. Working with ancient samples presents significant challenges due to scarce and degraded DNA and the risk of contamination. Reducing this risk requires strict precautions and careful authentication.

While aDNA research is traditionally performed in specialized laboratories, recent work has demonstrated that with rigorous contamination prevention and careful sample management, conventional forensic tools can produce reliable genetic results from degraded archaeological skeletal materials, offering meaningful insights into the study of past human populations.
